# Biomarkers and Microscopic Colitis: An Unmet Need in Clinical Practice

**DOI:** 10.3389/fmed.2017.00054

**Published:** 2017-05-10

**Authors:** Laura Francesca Pisani, Gian Eugenio Tontini, Beatrice Marinoni, Vincenzo Villanacci, Barbara Bruni, Maurizio Vecchi, Luca Pastorelli

**Affiliations:** ^1^Gastroenterology and Digestive Endoscopy Unit, IRCCS Policlinico San Donato, San Donato Milanese, Italy; ^2^Institute of Pathology, “Spedali Civili” Brescia, Brescia, Italy; ^3^Pathology and Cytodiagnostic Unit, IRCCS Policlinico San Donato, San Donato Milanese, Italy; ^4^Department of Biomedical Sciences for Health, University of Milan, Milan, Italy

**Keywords:** microscopic colitis, collagenous colitis, lymphocytic colitis, biomarkers, chronic diarrhea

## Abstract

One of the most common causes of chronic diarrhea is ascribed to microscopic colitis (MC). MC is classified in subtypes: collagenous colitis (CC) and lymphocytic colitis (LC). Patients with MC report watery, non-bloody diarrhea of chronic course, abdominal pain, weight loss, and fatigue that may impair patient’s health-related quality of life. A greater awareness, and concomitantly an increasing number of diagnoses over the last years, has demonstrated that the incidence and prevalence of MC are on the rise. To date, colonoscopy with histological analysis on multiple biopsies collected along the colon represents the unique accepted procedure used to assess the diagnosis of active MC and to evaluate the response to medical therapy. Therefore, the emerging need for less-invasive procedures that are also rapid, convenient, standardized, and reproducible, has encouraged scientists to turn their attention to the identification of inflammatory markers and other molecules in blood or feces and within the colonic tissue that can confirm a MC diagnosis. This review gives an update on the biomarkers that are potentially available for the identification of inflammatory activity, related to CC and LC.

## Introduction

**Figure 1:**
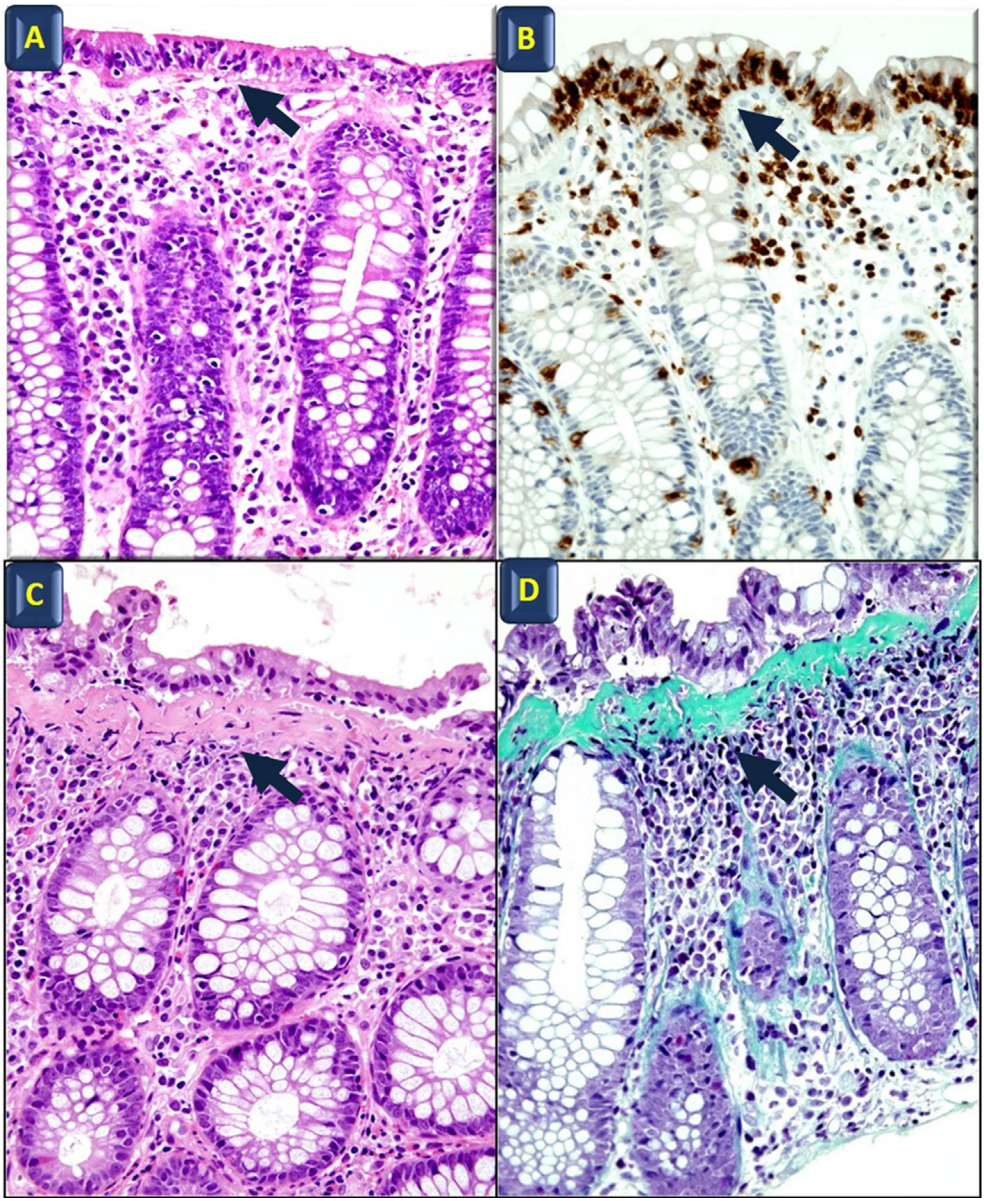
**Histological features of microscopic colitis**. Hematoxylin and eosin **(A)** and anti-CD3 **(B)** staining in lymphocytic colitis; black arrows indicate intraepithelial lymphocyte infiltration. Hematoxylin and eosin **(C)** and Masson’s Trichrome staining **(D)** in collagenous colitis; arrows point to the subepithelial collagen band. All images also show surface epithelial injury and lamina propria increased cellularity (original magnification: 40×).

Microscopic colitis (MC) is considered one of the most common causes of chronic, watery diarrhea in developed countries. Patients with MC have essentially a normal endoscopic appearance, with occasional erythema and/or edema patchy distributed along the colon (1–3). Lymphocytic colitis (LC) and collagenous colitis (CC) are the two main histological forms of MC (4). The histopathological hallmark of LC is a significant lymphocytic infiltration in the surface epithelium, and the diagnosis of LC is supported when the intraepithelial lymphocytes exceed 25 per 100 epithelial cells. Meanwhile, CC is defined by the presence of a subepithelial collagen band thicker than 10 μm. Both CC and LC show an intense infiltration in the lamina propria, mainly of T cells, but there are also plasma cells, eosinophils, mast cells, macrophages, and neutrophils (5, 6) (Figure 1) with a normal architecture of the crypts (5).

The pathogenesis of MC has yet to be fully clarified (7). Although both immune profiles and disease features remain heterogeneous among patients (6–9), CC and LC are currently thought to originate from a specific pathological response of the colonic mucosa to several luminal noxious agents in predisposed individuals, leading to an inappropriate immune response.

On a clinical point of view, both LC and CC present with very similar symptoms, such as chronic or relapsing watery non-bloody diarrhea, most likely of secretory origin. Less frequently, symptoms include cramping, abdominal pain, fecal incontinence, and weight loss. Together with the elimination of the chronic consumption of drugs, such as simvastatin, lansoprazole, and ticlopidine (10–12), budesonide, which is released in the terminal ileum and right colon, induces remission within several days. Conversely, treatment with immunosuppressive agents has no clear effect and relevant number of adverse events (1, 13, 14), even if immunosuppressive agents should be effective in patients who are corticosteroid-dependent or do not respond to corticosteroid treatment (15). Laboratory tests are usually normal or disclose unspecific abnormalities.

Previous reports have shown that the fecal biomarkers currently used to confirm diagnosis and to predict mucosal activity in inflammatory bowel diseases (IBDs) [i.e., fecal calprotectin (FC)] can be variably increased in some patients with CC (16). However, despite being primarily expressed by inflammatory cells, such as neutrophilic granulocytes, these biomarkers do not reflect the lymphocytic mucosal infiltration and their diagnostic accuracy is usually low in patients with MC.

Moreover, from a clinical point of view, there are no features that allow clinicians to distinguish between these two conditions and the other causes of chronic, watery diarrhea, including functional bowel disorders (17). Therefore, after having ruled out other causes of chronic, watery diarrhea with non-invasive laboratory tests (e.g., celiac disease, bowel parasitosis, thyroid dysfunction, exocrine pancreatic deficiency, and so forth) patients suspected for MC are invited to perform a complete colonoscopy, with several biopsies throughout the colon. In addition, besides being an invasive diagnostic tool, colonoscopy is often carried out to reassess the diagnosis in symptomatic patients with a history of MC, as well as to evaluate microscopic healing and response to therapy in clinical trials (18, 19).

Recent studies have focused on the need to reduce random colonic biopsies in patients with diarrhea, considering some characteristic features found in MC patients. Those with MC are more than 50 years of age, report weight loss, have a duration of diarrhea < 12 months, have recently taken a new drug, and have a coexisting autoimmune disease(s) (19). These risk factors can help identify those at higher risk of MC and identify those who should undergo random colonic biopsies (20). However, the sensitivity of these parameters is unacceptably low in terms of the potential loss of a MC diagnosis.

For these reasons, there is an emerging need to identify a non-invasive diagnostic tool that can predict the presence of active MC and distinguish MC from other functional and organic causes of watery diarrhea. Such a test would furthermore serve to better assess the real need for colonoscopy and histological evaluation. Ideally, this non-invasive diagnostic tool should be rapid, inexpensive, standardized, reproducible, and accurate in reflecting the predominant lymphocytic activity in the large bowel of patients suffering from MC.

The aim of this review is to provide an update on the issue of diagnostic biomarkers of MC; this overview highlights the emerging diagnostic tools that should improve our clinical practice in the near future.

## Epidemiology of MC

Microscopic colitis is an emerging disease. In fact, the number of patients diagnosed with MC has been increasing over the past 20 years (1). According to epidemiologic studies from Europe and US, the prevalence of MC likely ranges from 48 to 219 per 100,000 person-years (21).

The most recent meta-analysis performed by Tong et al. (22) showed that the overall incidence of CC was 4.14 (95% CI 2.89–5.40) per 100,000 person-years, while the incidence of LC was 4.85 (95% CI 3.45–6.25) per 100,000 person-years at risk.

Geographic variation in the incidence of MC has also been observed. The incidence of MC in a recent Danish population-based study was found to have markedly increased from 4.6 per 100,000 persons in 2002 to 24.7 per 100,000 persons by the end of 2011 (23). Similarly, a population-based study from Olmsted County, Minnesota found that incidence of MC increased from 1.1 per 100,000 persons in the late 1980s to 19.6 per 100,000 persons by the end of 2001 (24). A further population-based study conducted in the same region from 2002 to 2010 confirmed the previous trend, showing that the current incidence of MC is higher than initially expected, despite that, the growth appears to be less pronounced than before (25). The specific incidence of LC was 12.0 per 100,000 person-years (95% CI, 9.6–14.3 per 100,000 person-years) and CC was 9.1 per 100,000 person-years (95% CI, 7.0–11.1 per 100,000 person-years) (25). Epidemiological studies, most of them performed during the last decade, reported mean annual incidence rates from 1.8 to 5.4 per 100,000 person-years for CC and 1.3 to 4.5 per 100,000 person-years for LC (26–30). The causes of geographic variations are still unknown, but they probably arise from differences in background populations, environmental exposures, health-care systems, referral patterns, study designs, or diagnostic criteria.

The causes of the rise in incidence of MC are still a matter of debate and a recent study has identified that awareness of the disease and changes in the clinical behavior of endoscopists and pathologists may be the major drivers (31). It is still unclear whether this is just an epiphenomenon of an awareness-detection bias or whether this indicates a true increase of disease incidence, possibly driven by a wider exposure to some pathogenetic factors (e.g., drugs, environmental factors, nutritional factors).

## Increased Serum Proteins in the Setting of MC

At the present time, no reliable serum marker has been identified in MC. As a matter of fact, even though MC is sometimes associated with other immune/inflammatory diseases, it does not display prominent signs of systemic inflammatory activation. Thus, as previously stated, common serological inflammatory markers, such as C-Reactive Protein, are usually normal or only slightly elevated in both CC and LC (32) and, as a consequence, do not possess any significant role during the diagnostic phase. On the other hand, the prevalence of several autoantibodies is significantly higher in MC patients compared to controls. According to a recent manuscript from Roth et al., in a cohort of 133 Swedish women suffering from MC, the prevalence of anti-nuclear antibodies, anti-*Saccharomyces cerevisiae* IgG antibodies, anti-thyroid peroxidase, anti-perinuclear neutrophil cytoplasmic antibodies, and anti-glutamic acid decarboxylase was 14, 13, 14, 5, and 5%, respectively, with substantially lower values in control populations (5, 8, 7, 0, and 0%, respectively). In addition, when CC and LC were considered separately, the prevalence of these antibodies was greater in LC patients compared to CC (32). Another study from Holstein et al. found that 15% of patients with CC were positive for ASCA IgA and IgG, and 13% of those were also diagnosed with LC. However, the difference compared to the control group was statistically significant only for patients with CC. Positivity to ASCA should be interpreted as a non-specific epiphenomenon that should be generated from disturbances of the intestinal barrier (33). Regarding the study of autoantibodies in MC which also evaluates the levels of the anti-mitochondrial antibody, some research groups showed no variation in the levels of these antibodies, which are slightly increased in MC patients (34, 35). Despite the great interest of these data, it appears that none of the aforementioned antibodies have the potential to be used as a serological marker of MC, as they likely possess very low specificity and sensitivity, and they are probably linked to concomitant autoimmune diseases (32). Serum autoantibodies are further summarized in Table 1.

**Table 1:** **Prevalence of autoantibodies in serum of patients affected by microscopic colitis**.

Serum marker	Setting	Prevalence (%)	Sample size (*N*)	Reference
Anti-nuclear antibodies	CC	10	77	Roth et al. (32)
		26	26	Holstein et al. (33)
	LC	20	56	Roth et al. (32)
		12	16	Holstein et al. (33)
	HC	5	100	Roth et al. (32)
		5	43	Holstein et al. (33)
Anti-*Saccharomyces cerevisiae* IgG antibodies	CC	9	77	Roth et al. (32)
		15	26	Holstein et al. (33)
	LC	18	56	Roth et al. (32)
		13	16	Holstein et al. (33)
	HC	8	50	Roth et al. (32)
		0	43	Holstein et al. (33)
Anti-thyroid peroxidase	CC	12	77	Roth et al. (32)
	LC	16	56	Roth et al. (32)
	HC	7	50	Roth et al. (32)
Anti-perinuclear neutrophil cytoplasmic antibodies	CC	5	77	Roth et al. (32)
	LC	5	56	Roth et al. (32)
	HC	0	50	Roth et al. (32)
		1	43	Holstein et al. (33)
Anti-glutamic acid decarboxylase	CC	5	77	Roth et al. (32)
	LC	5	56	Roth et al. (32)
	HC	0	120	Roth et al. (32)
		0	43	Holstein et al. (33)
Anti-mitochondrial antibodies	CC	Not declared	13	Protic et al. (34)
	CC	8	38	Bohr et al. (35)
	LC	Not declared	46	Protic et al. (34)
	HC	Not declared	18	Protic et al. (34)
		5	38	Bohr et al. (35)

*LC, lymphocytic colitis; CC, collagenous colitis; HC, healthy control*.

## Increased Fecal Proteins in the Setting of MC

The identification of sensitive and sufficiently specific biomarkers in the feces of patients affected with MC hold promise of encouraging significant changes in the diagnostic flowchart. Indeed, fecal material is easy to collect and testing for fecal biomarkers may represent a screening tool before performing more invasive examinations, such as colonoscopy. Theoretically, in order to be sensitive and specific, fecal markers in MC have to recapitulate the most prominent histologic and biochemical feature of these diseases, which is the presence of an abundant intraepithelial lymphocytic infiltrate. Thus, molecules that ascertain the presence of abundant lymphocytic cellularity and that are not degraded within the stools might be promising candidates as MC fecal biomarkers. Unfortunately, at the present time, such markers do not exist, leaving the need for them completely unmet.

On the other hand, the diagnostic performances of some fecal markers of granulocyte activation have been tested in MC, even though granulocytes are definitely less prominently represented in MC pathology. Indeed, in clinical practice, several molecules originating from secreted granules of these cells are already widely used in identifying inflammatory conditions of the colon; as such, they have also to be tested in MC. Fecal markers are further summarized in Table 2.

**Table 2:** **Fecal proteins increased in MC**.

Cell type	Fecal marker	Setting	Findings and statistics	Sample size (*N*)	Reference
Neutrophils	Myeloperoxidase	CC vs. HC	Median 11.7 vs. 2.5 µg/g *p* < 0.05	18 vs. 20	Lettesjö et al. (36)
		CC vs. IBS	Median 11.7 vs. 1.7 µg/g *p* < 0.01	18 vs. 46	Lettesjö et al. (36)
		CC vs. HC	10.4 vs. 4.9 µg/g	9 vs. 45	Wagner et al. (37)
		LC vs. HC	9.6 vs. 4.9 µg/g	4 vs. 45	Wagner et al. (37)
	Calprotectin S100A8/S100A9	Active CC vs. Quiescent CC	Median 80 vs. 26 µg/g *p* = 0.025	21 vs. 12	Wildt et al. (16)
		CC vs. HC	Median 80 vs. 6.25 µg/g *p* = 0.002	21 vs. 13	Wildt et al. (16)
		IBD vs. other colitis^a^ vs. IBS	Median 349 vs. 92 vs. 49 µg/g *p* < 0.0001	24 vs. 21 vs. 21	Caviglia et al. (38)
		CC vs. HC	74 vs. 61 µg/g	9 vs. 45	Wagner et al. (37)
		LC vs. HC	42.7 vs. 61 µg/g	4 vs. 45	Wagner et al. (37)
	Lactoferrin	Active CC vs. Quiescent CC	1 vs. 0 (no. of positive tests)	21 vs. 12	Wildt et al. (16)
Eosinophils	Eosinophil protein X	CC vs. HC	Median 3.8 vs. 0.46 µg/g *p* < 0.001	18 vs. 20	Lettesjö et al. (36)
		CC vs. IBS	Median 3.8 vs. 0.44 µg/g *p* < 0.001	18 vs. 46	Lettesjö et al. (36)
		CC vs. HC	5.7 vs. 0.82 µg/g *p* = 0.01	9 vs. 46	Wagner et al. (37)
		LC vs. HC	1.7 vs. 0.82 µg/g	4 vs. 46	Wagner et al. (37)
	Eosinophil cationic protein	CC vs. HC	92% of CC > upper limit of normal	12 vs. 44	Wagner et al. (39)
		CC vs. HC	5.3 vs. 1.5 µg/g *p* = 0.01	9 vs. 46	Wagner et al. (37)
		LC vs. HC	2.6 vs. 0.82 µg/g	4 vs. 46	Wagner et al. (37)
Mast cells	Tryptase	CC vs. IBS vs. HC	50 vs. 13 vs. 5.3% detectable levels	18 vs. 46 vs. 19	Lettesjö et al. (36)
Other leukocytes	IL-1β	CC vs. IBS vs. HC	18% CC detectable levels Undetectable in IBS and HC	18 vs. 46 vs. 19	Lettesjö et al. (36)
	Tumor necrosis factor α	CC vs. IBS vs. HC	Undetectable levels	18 vs. 46 vs. 19	Lettesjö et al. (36)
Enteroendocrine cells	Chromogranin A	CC vs. HC	*p* < 0.001	12 vs. 43	Wagner et al. (40)
		CC vs. IBD	*p* < 0.001	12 vs. 32	Wagner et al. (40)
	Chromogranin B	CC vs. HC	*p* < 0.001	12 vs. 43	Wagner et al. (40)
		CC vs. IBD	*p* < 0.001	12 vs. 32	Wagner et al. (40)
	Secretoneurin	CC vs. HC	*p* < 0.01	12 vs. 43	Wagner et al. (40)
		CC vs. IBD	*p* < 0.001	12 vs. 32	Wagner et al. (40)

*LC, lymphocytic colitis; CC, collagenous colitis; HC, healthy control; IBD, inflammatory bowel disease; IBS, irritable bowel syndrome*.

*^a^Other colitis: microscopic colitis (MC), eosinophilic colitis, and non-specific chronic colitis*.

### Fecal Proteins of Neutrophilic Origin

Most of the data available are related to neutrophil granulocyte proteins. More specifically, neutrophils can release a multiplicity of toxic oxygen radicals and a variety of granular and soluble proteins. Neutrophils play an important defensive role against bacteria. Nonetheless, they are also believed to cause mucosal tissue injury, leading to the development of several inflammatory conditions of the bowel, including MC. The levels of neutrophil-derived myeloperoxidase (MPO), a lysosomal peroxidase with a powerful antimicrobial activity, are usually increased in patients with active CC, supporting the role of neutrophils in this pathology, and not only in IBD. On the contrary, MPO levels in patients with irritable bowel syndrome (IBS) did not differ from healthy controls (36, 39). Calprotectin, also known as S100A8/S100A9 complex (41), is a calcium-binding protein with antibacterial, anti-proliferative, and immunomodulating effects. It constitutes two-third of the cytosolic proteins stored into neutrophilic granulocytes, even though smaller amounts of this protein are also present in macrophages and monocytic cells (42). Previous studies have clearly shown that FC levels are directly proportional to the neutrophil migration through the gut wall and increase with the severity of inflammation (38). Consequently, this protein is significantly increased in the feces of patients with active inflammatory processes in which neutrophilic infiltration in the large-bowel mucosal layer is consistent, such as in ulcerative colitis (UC) and Crohn’s colitis. Recently, the associations between FC and histological inflammation, disease activity indices (43, 44), and the ability to predict a potential relapse in IBD have been deeply described in UC (45).

Wildt et al. have clearly showed that FC is significantly increased in CC patients as compared to healthy controls, demonstrating a significant difference between patients with active and quiescent disease (16). This evidence confirmed that the mucosal inflammatory process in patients with CC is in part due to the activation of neutrophils. However, such a pathologic increase in FC was not a universal finding; some patients with active CC had normal FC and its concentrations may differ considerably in patients with active disease (16). More specifically, this study showed that out of 21 CC patients with active disease, only 13 presented with an increased FC (16). To date, it is unclear if this difference depends on immunologic responses or on different degrees of inflammation. As a matter of fact, both the frequency and the entity of FC increase are quite variable, although more consistent in UC and also CD—disease conditions in which the role of FC is more established.

Lactoferrin is another protein contained in neutrophil granules, which is released during active inflammatory conditions (46). Fecal lactoferrin (FL), as is calprotectin, is a marker of intestinal inflammation (46). In 1998, Fine et al. tested the increase of FL concentrations in the feces of 103 patients affected by chronic diarrhea of unknown etiology and compared it to 10 healthy control subjects as well as patients with an already known intestinal disease (8 Crohn’s disease; 4 UC; 13 celiac disease; 26 MC) (47). FL results were normal in all healthy subjects and was elevated in all previously diagnosed IBD patients as well as in three MC patients. Among the 103 patients with chronic diarrhea, 11 presented with increased FL. Patients underwent extensive valuation including endoscopic biopsies of the colon and small intestine before the final diagnoses for the latter group were made and correlated with FL testing results: 10 patients had IBD and FL presented with elevated values in 9 of them; 1 patient had an ischemic colitis together with high FL; MC was revealed in 13 patients, but only 1 presented elevated FL. Overall, whereas FL was highly accurate in confirming or identifying IBD (90% sensitivity, 98% specificity), only a small percentage of MC patients (4 out of 39, 10%) showed positive FL (47). The prevalence of FL in CC was also evaluated also in the aforementioned work from Wildt et al. (16). In this study, only 1 patient had increased FL concentrations in stools, suggesting a very low sensitivity of the test in this setting (16).

Overall, it appears that, in a significant proportion of MC patients, allegedly those with less severe histologic activity, secretory granules proteins are not highly represented in stools; therefore, the role of those molecules as markers of MC seems to be limited.

### Fecal Proteins Produced by Eosinophils

Eosinophilic granulocytes are potent pro-inflammatory cells: they can induce epithelial damage by releasing several cytotoxic granule-derived proteins (48). In addition, they secrete other mediators, such as cytokines, chemokines, and leukotrienes, which modulate epithelial inflammation (6). In 2001, Levy et al. demonstrated that eosinophilic infiltration and degranulation were remarkably increased in CC patients’ colonic mucosa as compared to healthy controls (49). Consistently, two further pilot studies have shown that eosinophil-related inflammatory markers, such as eosinophil cationic protein (ECP) and eosinophil protein X (EPX), are significantly increased in fecal samples from CC patients as compared to those of IBS patients (36, 39). Remarkably, a very recent study tested the diagnostic performance of fecal ECP and EPX, together with FC and fecal MPO, in a cohort of 67 patients referred to colonoscopy due to chronic non-bloody diarrhea (37). This specific setting is very important, since it recapitulates the clinical scenario where the availability of MC biomarkers could represent a step forward in clinical practice. According to endoscopy and pathology reports (available for 63 out of 67 patients), 46 patients were affected with IBS; 2 with UC; 2 with CD; 2 with LC; and 9 with CC. The results of this study are very promising; in fact, fecal ECP and EPX were significantly higher in CC patients and when all the four tested fecal markers (ECP, EPX, FC, and MPO) were negative, the chances of getting a normal histological description were 92%. In addition, in the same study, serum levels of ECP and EPX were also measured, without finding any differences between the different study groups (37). Indeed, given the small number of patients included, these results need to be confirmed in larger cohorts, but they still suggest a potential usefulness of eosinophil-related inflammatory molecules as markers of MC, and more specifically of CC. It is important to keep in mind that eosinophils are also indicative of drug-induced inflammation, beyond CC and LC (50).

### Fecal Proteins from Other Leukocytes

Molecules produced by other types of inflammatory cells have been considered, as well. The mast cells are located in the mucosa of different organs and tissues and are the main actors in allergic reactions. The role of mast cells in inflammatory conditions of the gut is not completely understood yet; however, it is known that mast cells have an increased propensity to release tryptase in UC (51). The presence of this enzyme in fecal matter was also studied in CC patients, and detectable levels of fecal tryptase were present in about 50% of CC patients, as compared to 13 and 5% of IBS patients and healthy controls, respectively (36).

In the same paper, Lettesjö et al. also measured the fecal levels of two potent pro-inflammatory molecules, those being interleukin 1β (IL-1β) and tumor necrosis factor (TNF)-α. These cytokines are produced and released primarily by mucosal macrophages but also by many different inflammatory and non-inflammatory cell types. Indeed, their role and overexpression in active IBD is well established (52–56), thus suggesting the rationale to test them in MC. Despite this premise, only 18% of patients affected by CC presented with enhanced fecal IL-1β levels, while levels in the other experimental groups were very low or undetectable, and TNF was not detectable in any stool sample (36).

### Fecal Proteins from Neuroendocrine Cells

The clinical presentation of MC presents as an increased secretory activity of the bowel and decreased absorption of water and electrolytes. The neuroendocrine system of the gut regulates gut motility, furthermore guiding water and salts absorption (57–59).

Thus, El-Salhy et al. hypothesized that in MC there may be a hyperactivation/hypertrophy of colonic neuroendocrine cells, and consequently, in their study, the authors demonstrated by immunohistochemistry an overabundance of Chromogranin A (CgA)^+^ cells interspersed among epithelial cells in LC (no data are provided for CC) (60, 61). CgA is part of the granin family and a marker for enteroendocrine cells, a protein which is elevated in plasma and serum of IBD patients (62). In addition, in most neuroendocrine cells, Chromogranin B (CgB) coexists with CgA, and it can be used complementary with CgA as an important marker for detecting neuroendocrine tumors (63, 64). Also, secretoneurin (SN) is a major peptide within the human enteric neuroendocrine system (65) and it is a chemoattractant for blood eosinophils (66), which also increases spontaneous locomotion of neutrophils (67). Therefore, Wagner et al. measured these molecules in the feces of CC patients and they found that CgA, CgB, and SN are detectable in CC patients’ feces and are also markedly overexpressed when compared to healthy controls and patients with IBD. The considerable differences in the expression of these neuropeptides between groups suggest that the enteric nervous system is clearly involved in the pathophysiology of CC (40). Levels of CgA and CgB are persistently high during treatment in CC patients; on the contrary, after therapy, SN levels decrease and reach levels found in healthy controls, suggesting an upregulation of the enteroendocrine system (40). In LC patients, CgA has never been measured in feces (60). While fecal CgA, CgB, and SN are possibly promising markers, studies aimed at evaluating prevalence, the best thresholds, sensitivity and specificity in predicting MC are strongly needed.

## Histological Markers

The diagnosis of MC is mostly based on histological assessment. Therefore, several markers have been studied using immunohistochemistry to distinguish between CC and LC and, thus, clarify the diagnosis when hematoxylin and eosin staining is not sufficient, as may be the case of “incomplete MC” (iMC). This term refers to patients who have the clinical presentation of MC but partially fulfill the characteristic histological criteria for the diagnosis of LC and CC. In particular, in iMC patients, an increase in the number of IELs is observed, but <20/100 epithelial cells in superficial epithelium (incomplete LC) and abnormal thickening of the subepithelial collagen band <10 µm (incomplete CC). The inflammatory infiltrate in the lamina propria is also increased. These conditions are classified in literature as borderline LC, minimal CC, MC not otherwise specified, and paucicellular LC. The term “undefined MC” was chosen to avoid any confusion with the iMC term and was introduced with the following definition: “no information for further subtyping was available.” From a strictly histological point of view, however, only the terms CC and LC are advisable, and the use of the above-mentioned definitions should be avoided when preparing a pathological report (68).

Langner et al. (5) recently reviewed stainings that are commonly used in clinical practice. Anti-CD3 identifies lymphocytes, thereby improving the diagnosis of LC. Anti-CD68 identifies cells belonging to the monocyte/macrophage lineage and may be useful in identifying LC and CC with giant cells (variants characterized by the presence of multinucleated giant cells originating from the fusion of subepithelial macrophages) (69–71). Finally, Masson’s trichrome stain highlights the presence of the collagen band in CC (5) (Figure 1). Notably, the thickness of the subepithelial collagenous band-like deposit usually shows a remarkable variation throughout the colon in CC. In addition, routine staining (i.e., hematoxylin and eosin, Masson’s trichrome) do not selectively highlight these deposits (72). The solution to this problem would be to label those extracellular matrix (ECM) components, which are specifically expressed in the subepithelial collagen band.

The abnormal collagen deposition seems to originate from disturbances in the function of subepithelial myofibroblasts, resulting in increased synthesis or decreased degradation of ECM (73). These cells contribute to the maintenance of the intestinal mucosa structure and can synthetize several ECM matrix components (73). Tenascins (TNs) are a family of large ECM glycoproteins, which are involved in cell adhesion and migration during development, tissue homeostasis, and responses to disease or trauma. Many TNs can influence the way that fibronectin signals through integrins, they have the ability to signal directly through integrin receptors or by binding to the ECM glycoprotein fibronectin (74). In normal mucosa, in non-specific chronic inflammation, and in LC, TNs are weakly expressed; immunostaining detects TNs in the majority of cases defined as “not detectable” following standard trichrome stains, even though a strong subepithelial positive band with lacy appearance and coarse prolongation into the lamina propria was observed (73, 75). In CC, a prominent extension of the collagen band measuring 12–28 µm was detected using TNs immunostaining, resulting in a more accurate measurement of the thickness of the subepithelial collagen deposition (76). Immunohistological staining for TNs could be used as a routine approach in cases of clinically suspected CC. Other ECM components, such as laminin and fibronectin, were not uniformly expressed and distributed in the lamina propria of normal and inflamed tissues, giving them no value as diagnostic markers of MC (73).

Recent studies on fecal stream diversion proposed a model in which the collagen deposition is reversible (77, 78). Immunohistological findings and *in situ* hybridization analyses on the composition of the immediate pericryptal ECM suggest that the pericryptal myofibroblasts express minor amounts of collagen type I, III, and VI in the deep parts of the crypts (79). In the upper pericryptal area and particularly in the sub-epithelium, cells express and deposit increased amounts of type VI collagen and initiate TNs synthesis, while some type III collagen is also detectable (80). The fact that both proteins accumulate within the band-like structures suggests that they represent a pathological accumulation of physiological products of the subepithelial myofibroblasts (75).

The excessive collagen deposition seems to reflect a local disturbance in ECM turnover, resulting in the formation of a provisional ECM. Among ECM-degrading enzymes, matrix-metalloproteinases (MMPs) have a central role. MMPs are a family of zinc-dependent neutral proteinases with overlapping but distinct substrate spectra. Four subfamilies of MMPs are known: collagenases, gelatinases, stromelysins, and membrane-MMPs (81, 82). Together with MMP-8 (collagenase-2, neutrophil collagenase), MMP-1 (collagenase-1, interstitial collagenase) and MMP-13 (collagenase-3) form the MMP-subfamily of collagenases, which are instrumental for the degradation of native interstitial collagens, in particular collagen type I, II, and III (81, 82). Among the abovementioned MMPs, only MMP-1 seems to have a role in CC and its expression is increased in subepithelial myofibroblasts (83). In any case, MMP-1 expression is counteracted by increased tissue of metalloproteinases (TIMP)-1 inhibitor, showing a local impairment in ECM degradation in CC (83).

Vascular endothelial growth factor (VEGF) is a potent pro-angiogenic molecule, which also increases vascular permeability and is overexpressed during growth and metastasis in tumors. In chronic inflammatory disorders, it promotes tissue repair and plays a central role in ECM degradation (84–86). VEGF limits the release of TIMP-1 by endothelial cells, thereby increasing MMP-1 activity (16, 45). In the epithelium and in inflammatory cells of the lamina propria, expression of VEGF reflects an important physiological mechanism counteracting the MMP-1/TIMP-1 imbalance in CC; disinhibiting MMP-1 and reducing levels of TIMP-1 leads to an accumulation of immature subepithelial ECM in CC (87). An increased immunostaining for VEGF within the epithelium is also maintained when clinical and histological remission is achieved, suggesting the persistence of the repair mechanism (87).

Several studies have been performed on arachidonic acid metabolism. The main roles described for prostaglandin (PG) in the gastrointestinal tract are related to the enhancement of barrier function, wound repair and restitution following damage, regulation of mucosal blood flow, and also mucus production and secretion.

Prostaglandins are also involved in immune function regulation. A case report (88) demonstrated that a patient with CC had extremely high luminal excretion of PGE2 as compared to a control subject; PGE2 exerts pro-inflammatory activity by upregulating IL-8 in colonic epithelial cells. Another study found that PGE2 increases the inflammatory response in the bowel through EP4 receptors in dendritic cells (89) and decreases the production of inflammatory cytokines in intestinal T cells (90). Elevated EP4 receptor levels correlate with high levels of TNF-α and could be studied as an ideal biomarker for MC (91).

Cyclooxygenase (COX) is an enzyme involved in PG synthesis *via* the arachidonic acid pathway and is present in two isoenzymes: COX-1 and COX-2. The constitutively expressed isoenzyme is COX-1, which is believed to be responsible for the production of PGs associated with gastrointestinal integrity. Whereas COX-2 is an inducible isoenzyme, it is rapidly induced by a variety of stimuli, such as cytokines, growth factors, hormones, and carcinogens, and can also induce the production of PGs contributing to inflammation (92). By using western blot analysis, immunohistochemistry, and immunofluorescence methods on biopsies from patients with CC, the presence and cellular localization of COX-2 in colonic mucosa of patients with CC was shown to be increased both quantitatively and qualitatively (93). The cellular distribution of COX-2 was observed in the inflammatory infiltrate of the lamina propria, in both plasma cells and particularly in the macrophagic subpopulation of the mononuclear cells (93). Enhanced expression of COX-2 was shown in the lamina propria of biopsies from patients with IBD at intensities similar to CC, given this COX-2 should not be useful as a biomarker for MC.

The free radical gas, nitric oxide (NO) is a mediator of inflammation, which may exert secretory actions in the human colon (94). In the normal colon, NO is produced from the amino acid l-arginin in epithelial cells, endothelial cells, and submucosal neuronal cells by constitutive NO synthase (95). In addition, normal colonic epithelial cells express the inducible isoform of NOS, iNOS, which may contribute to normal mucosal barrier function (96). As observed in ulcerative, infectious, collagenous or lymphocytic colitis, the generation of NO is highly increased (97–99). In biopsy specimens from patients with chronic diarrhea and “minimal colitis,” the iNOS antibody was observed only at the luminal border in the crypt epithelium, while in healthy volunteers it was localized in the epithelial cells of the colon. Western blot analysis showed significantly higher iNOS in symptomatic patients when compared to healthy volunteers (100). The results of these studies suggest that patients with chronic, idiopathic diarrhea have an upregulation of iNOS activity in colonic mucosa, which results in a severe impairment of fluid absorption in the colon.

Taken together, several potential markers for the histopathological diagnosis of MC do exist, but their capacity in predicting the presence of this disease still needs to be confirmed and measured in clinical settings. Indeed, given the high accuracy of histologic findings in identifying CC and LC, it is questionable whether tissue markers may really improve the ability to diagnose MC in clinical practice. As a matter of fact, a possible use of these markers may be in those MC cases with incomplete histologic or borderline features. In addition, it is worth pointing out that some histologic markers may have the potential to limit the invasiveness of the endoscopic procedure. In fact, we previously mentioned that El-Salhy et al. showed a markedly higher density of CgA-expressing cells in LC colonic mucosa, as compared to non-MC controls in two studies (61). Interestingly, the second study not only confirmed these results but also demonstrated that the intense increase of CgA^+^ cells in LC was consistently present both in right and left colonic biopsies (60). As such, the authors speculated that, if confirmed, this finding may permit to differentiate between a diagnosis of LC and IBS by collecting biopsies during a simple sigmoidoscopy, without the need to perform a complete colonoscopy. Indeed, this may represent a great advantage for patients who have already undergone full colonoscopy without tissue sampling during the initial diagnostic work-up or before the onset of diarrhea. Tissue markers are further summarized in Table 3.

**Table 3:** **Tissue markers in microscopic colitis (MC)**.

Tissue marker	MC	Localization	Diagnostic accuracy	Sample Size (*N*)	Reference
Chromogranin A	LC	Higher in left colon	27.2 ± 1.4 cells/mm^2^ LC vs. 8.9 ± 0.6 cells/mm^2^ HC	57 vs. 54	El-Salhy et al. (60)
Tenascins (TNs)	CC CC	Subepithelial band Subepithelial band	12–28 µm CC vs. 4–6 µm HC 29.46 ± 1.87 μm CC vs. no TN HC	15 vs. 15 35 vs. 18	Anagnostopoulos et al. (76) Salas et al. (73)
Type IV collagen	CC	Deep part of the crypts, penetrating blood vessels	CC > 10 labeled cells/mf	12 vs. 7	Günther et al. (83)
Matrix-metalloproteinase-1	CC	Subepithelial band	CC > 7 labeled cells/mf	12 vs. 7	Günther et al. (83)
Tissue of metalloproteinases-1	CC	Subepithelial band	CC > 10 labeled cells/mf	12 vs. 7 21 vs. 5	Günther et al. (83) Griga et al. (87)
Vascular endothelial growth factor (VEGF)	CC	Epithelium	11.61% CC vs. 1.10% HC	21 vs. 5	Griga et al. (87)
VEGF	CC	Leukocytes in the Lamina propria	0.89% CC vs. 0.04% HC	21 vs. 5	Griga et al. (87)
EP4 receptor	CC	Intestinal epithelial cells, lymphocytes, and lamina propria	expression CC > 10 folds vs. HC	8 vs. 12	Dey et al. (91)
Cyclooxygenase-2	CC	Mononuclear cells in the lamina propria	Staining ratio 1.93 CC vs. 2.59 HC	10 vs. 8	Wildt et al. (93)
iNOS	MC	Epithelial cells of the luminal border of crypts	OD 8.2 ± 1.5 MC vs. OD 0.8 ± 0.2 HC	12 vs. 6	Perner et al. (100)

*LC, lymphocytic colitis; CC, collagenous colitis; HC, healthy control; mf, microscopic field; OD, optical density*.

## Conclusion

Over the last few years, growing evidence has shed new light on the pathogenesis of MC and has increased physicians’ awareness, thus resulting in several concrete improvements in the clinical management of these emerging disorders. To date, no reliable biomarker of CC and LC is available, and histologic examination based on multiple biopsies performed during colonoscopy is still the unique tool used to assess the diagnosis in patients with suspected MC.

In order to overcome such limitations and to encourage more patient-friendly and cost-saving policies, an increasing number of studies are amassing a wealth of data on different putative serological and fecal tests for MC. Although newly identified markers hold some potential, so far, none of the tested molecules present sufficient accuracy for use in clinical practice, appearing as more useful to study MC pathogenic mechanisms, rather than to predict disease activity. In particular, none of the currently available non-invasive tests allows clinicians to screen and monitor patients affected with CC and LC, in order to perform endoscopic and pathological evaluations only in selected cases. We speculate that, similar to what happens for FC, reflecting neutrophil mucosal infiltration in UC, the ideal biomarker for MC should mirror CD3^+^ lymphocyte collection in the mucosal/epithelial layer, thereby showing high sensitivity and specificity in distinguishing MC from either healthy subjects or other forms of IBDs. Limited by low prevalence and heterogenic disease phenotypes, the identification of such biomarkers appears, indeed, to be a challenging task in MC. Nonetheless, another key issue, which reduces the reliability of the few available data, is that most of the clinical studies performed thus far include only patients already possessing a confirmed diagnosis of MC. In fact, the identification and development of biomarkers with high sensitivity and specificity should test how those markers perform in unselected patients in the specific clinical setting; hopefully, more studies will be planned mirroring the real-life clinical scenario, in which biomarkers are needed, as was the case for fecal ECP and EPX (37). As long as non-invasive testing remains an unmet need, all patients suspected for active MC will be directly referred to colonoscopy with extensive histopathological evaluation and this will continue to have a predictable impact on both everyday clinical practice and patients’ perspectives.

## Author Contributions

LFP, GT, BM, and LP wrote the paper; VV provided histological images; VV, BB, MV, and LP critically revised the paper.

## Conflict of Interest Statement

The authors declare that the research was conducted in the absence of any commercial or financial relationships that could be construed as a potential conflict of interest.
