# Corrigendum: Biomarkers and Microscopic Colitis: An Unmet Need in Clinical Practice

**DOI:** 10.3389/fmed.2020.00004

**Published:** 2020-01-31

**Authors:** Laura Francesca Pisani, Gian Eugenio Tontini, Beatrice Marinoni, Vincenzo Villanacci, Barbara Bruni, Maurizio Vecchi, Luca Pastorelli

**Affiliations:** ^1^Gastroenterology and Digestive Endoscopy Unit, IRCCS Policlinico San Donato, San Donato Milanese, Italy; ^2^Institute of Pathology, “Spedali Civili” Brescia, Brescia, Italy; ^3^Pathology and Cytodiagnostic Unit, IRCCS Policlinico San Donato, San Donato Milanese, Italy; ^4^Department of Biomedical Sciences for Health, University of Milan, Milan, Italy

**Keywords:** microscopic colitis, collagenous colitis, lymphocytic colitis, biomarkers, chronic diarrhea

In the original article, there was a mistake in the legend for **Figure 1** as published as Villanacci et al. (2011) was inadvertently not cited.

**FIGURE 1** | Histological features of microscopic colitis. Hematoxylin and eosin **(A)** and anti-CD3 **(B)** staining in lymphocytic colitis; black arrows indicate intraepithelial lymphocyte infiltration. Hematoxylin and eosin **(C)** and Masson's Trichrome staining **(D)** in collagenous colitis; arrows point to the subepithelial collagen band. All images also show surface epithelial injury and lamina propria increased cellularity (original magnification: 40×). This figure was obtained from Villanacci et al. (101) with permission.

The authors apologize for this error and state that this does not change the scientific conclusions of the article in any way. The original article has been updated.
